# Brace treatment in an infantile/juvenile patient with progressive scoliosis due to Marfan’s syndrome

**DOI:** 10.1186/1748-7161-7-S1-P5

**Published:** 2012-01-27

**Authors:** HR Weiss, M Werkmann

**Affiliations:** 1 Orthopedic Rehabilitation Services, Gensingen, Germany

## Background

Little information exists about successful brace treatment of progressive early onset scoliosis. Even less information is available about the early treatment of scoliosis patients with Marfan’s syndrome at age < 6 years. Purpose of this case report is to demonstrate the possibility of successful brace treatment in a patient with early onset scoliosis due to Marfan’s syndrome [1,2].

## Case report

A two year old girl diagnosed with Marfan’s syndrome presented with a double major scoliosis of 20°. After a follow-up of 6 months she showed a rapid progression to 46° (November 2008) and was braced immediately. In-brace correction in the first brace (RSC) was moderate due to the stiffness mainly of the lumbar curve. A new brace was made after significant growth (Gensingen brace in October 2009). An in-brace correction to 12° thoracic and 12° lumbar has been achieved. In October 2010 she also has outgrown her second brace to some extent. Due to clinical overcorrection (ATR lumbar -5°) brace wearing time has been reduced to 12 hrs. / day at first. In January 2011 at the age of 4 and a half she presented again with an ATR lumbar of -6° and thoracic 2°, lumbar still overcorrected, so we decided to leave off the brace for 3 months time.

## Conclusions

(1) Successful brace treatment in infantile / juvenile patients with scoliosis is possible. (2) When treated during periods of rapid growth corrections can be achieved with high correction braces. (3) Before early surgery is performed high quality conservative management seems indicated.
